# International core outcome set for clinical trials of medication review in multi-morbid older patients with polypharmacy

**DOI:** 10.1186/s12916-018-1007-9

**Published:** 2018-02-13

**Authors:** Jean-Baptiste Beuscart, Wilma Knol, Shane Cullinan, Claudio Schneider, Olivia Dalleur, Benoit Boland, Stefanie Thevelin, Paul A. F. Jansen, Denis O’Mahony, Nicolas Rodondi, Anne Spinewine

**Affiliations:** 1 Louvain Drug Research Institute (LDRI), Clinical pharmacy research group, Université catholique de Louvain, Brussels, Belgium; 2 Université Lille, EA 2694 - Santé publique: épidémiologie et qualité des soins, F-59000 Lille, France; 3 Department of Geriatric Medicine and Expertise Centre Pharmacotherapy in Old Persons, University Medical Centre Utrecht, Utrecht, The Netherlands; 4 Pharmaceutical Care Research Group, School of Pharmacy, Cavanagh Pharmacy Building, University College Cork, College Road, Cork, Ireland; 5 School of Pharmacy, Royal College of Surgeons in Ireland, Dublin, Ireland; 6 Department of General Internal Medicine, Bern University Hospital, Bern, Switzerland; 7 Pharmacy department, Cliniques universitaires Saint-Luc, Université catholique de Louvain, Brussels, Belgium; 8 Geriatric Medicine, Cliniques universitaires Saint-Luc, Institut de Recherche Santé et Société, Université catholique de Louvain, Brussels, Belgium; 9 Department of Geriatric Medicine, Cork University Hospital and Department of Medicine, University College Cork, Cork, Ireland; 10 Institute of Primary Health Care (BIHAM), University of Bern, Bern, Switzerland; 11 Pharmacy department, CHU UCL Namur, Université catholique de Louvain, Yvoir, Belgium

**Keywords:** Core outcome set, Medication review, Older patients, Multi-morbidity, Polypharmacy, Delphi survey, Consensus

## Abstract

**Background:**

Comparisons of clinical trial findings in systematic reviews can be hindered by the heterogeneity of the outcomes reported. Moreover, the outcomes that matter most to patients might be underreported. A core outcome set can address these issues, as it defines a minimum set of outcomes that should be reported in all clinical trials in a particular area of research. The objective in this study was to develop a core outcome set for clinical trials of medication review in multi-morbid older patients with polypharmacy.

**Methods:**

Firstly, eligible outcomes were identified through a systematic review of trials of medication review in older patients (≥65 years) and interviews with 15 older patients. Secondly, an international three-round Delphi survey in four countries involving patients, healthcare professionals, and experts was conducted to validate outcomes to be included in the core outcome set. Consensus meetings were conducted to validate the results.

**Results:**

Of the 164 participants invited to take part in the Delphi survey, 150 completed Round 1, including 55 patients or family caregivers, 55 healthcare professionals, and 40 experts. A total of 129 participants completed all three rounds. Sixty-four eligible outcomes were extracted from 47 articles, 32 clinical trial protocols, and patient interviews. Thirty outcomes were removed and one added after Round 1, 18 outcomes were removed after Round 2, and seven after Round 3. Results were discussed during consensus meetings. Consensus was reached on seven outcomes, which constitute the core outcome set: drug-related hospital admissions; drug overuse; drug underuse; potentially inappropriate medications; clinically significant drug-drug interactions; health-related quality of life; pain relief.

**Conclusions:**

We developed a core outcome set of seven outcomes which should be used in future trials of medication review in multi-morbid older patients with polypharmacy.

**Electronic supplementary material:**

The online version of this article (10.1186/s12916-018-1007-9) contains supplementary material, which is available to authorized users.

## Background

Patients aged 65 and older are often exposed to polypharmacy in a multi-morbidity context [1, 2]. This increases medication costs and the risk of adverse drug reactions [3–6]. Structured medication review has been shown to be an efficient way to optimize prescribing for older patients [7, 8]. Its impact on clinical, patient-reported, and economic outcomes has been evaluated in a wide range of randomized controlled trials (RCTs). Also, several systematic reviews and meta-analyses have been conducted to assess its effectiveness in various settings [7–19]. The quality of conclusions has, however, been limited by the heterogeneity of outcomes, among other factors. Robust meta-analyses could be performed for only a few outcomes, including hospitalization and death [14–16, 20].

A core outcome set (COS) defines a minimum set of outcomes to be reported in all clinical trials in a particular research area. The COS can (1) reduce heterogeneity between trials, (2) lead to research that is more likely to measure relevant outcomes, (3) enhance the value of evidence synthesis by reducing the risk of outcome reporting bias, and (4) ensure that all trials report usable information [21–23]. Outcome reporting bias is an underrecognized problem that affects conclusions in many systematic reviews [24–26]. Moreover, outcomes that are highly relevant to older adults are often ignored in RCTs [27, 28].

This work, part of the European OPtimising thERapy to prevent Avoidable hospital admissions in the Multimorbid elderly (OPERAM) project, aimed to develop a COS for clinical trials of medication review in older patients with multi-morbidity and polypharmacy.

## Methods

### Study design

The Outcome Measures in Rheumatology (OMERACT), Core Outcome Measures in Effectiveness Trials (COMET), and Core Outcome Set-STAndards for Reporting (COS-STAR) guidelines were used for developing and reporting this COS [21, 29, 30]. The scope of the COS was ’Medication review among patients aged 65 years and older with polypharmacy (≥5 daily medications) and multi-morbidity (≥2 chronic morbidities)’. The project was registered on the COMET database (http://www.comet-initiative.org/studies/details/806?result=true). Details on the study protocol have been published elsewhere [31]. The COS-STAR checklist is detailed in Additional file 1: Table S1.

The study was performed in four countries (Belgium, Ireland, Switzerland, and the Netherlands). The medical centers included tertiary academic medical centers with a wide range of surgical and internal medicine specialties, including geriatric medicine (Belgium, Switzerland, and the Netherlands), and a secondary medical center specialized in geriatrics (Ireland). A steering committee was set up with researchers from the four participating medical centers.

### Ethical approval

Ethical approval was obtained in Belgium and Ireland. In the Netherlands and Switzerland, official ethical approval was not required, as the ethics committees confirmed that the relevant legislation was not applicable.

### List of potential outcomes for inclusion in Delphi survey

As a first step, a systematic review identified all outcomes previously used or planned to be used for evaluating the effect of medication review among older patients. It was achieved through an update of a recent systematic review on medication review published by Lehnbom et al. [9] combined with a systematic search in randomized clinical trials registries and on the Cochrane Database. All data extractions on outcomes were performed by two independent reviewers. Any disagreement was resolved by discussion and consensus. Details have been published elsewhere [32].

In parallel, a qualitative study identified unknown and relevant outcomes that should be included in the Delphi survey. We held semi-structured one-to-one interviews with patients and family caregivers. Patients aged ≥ 65 taking at least five different daily medications were recruited from the geriatric outpatient clinic, the acute geriatric ward, and other medical and surgical wards in two Belgian teaching hospitals (purposive sampling). Family caregivers assisting such patients were eligible for inclusion. The interviews took the form of a discussion about the patient’s medications, the perception of risks and benefits of his/her medications, the concept of medication review, and what he/she would expect from a medication review. A topic guide was developed, pilot-tested, and used by both interviewers (AS and OD). Interviews were recorded and then transcribed verbatim. Audio recordings of the interviews were analysed using NVivo10®. Two independent researchers (a physician and a psychologist) used an interpretative approach to identify outcomes and outcome definitions based on participants’ descriptions. The analysis focused on identifying a list of outcomes that are important to older people. Analysts proceeded with coding, labelling, and indexing of data to facilitate the process of identifying relevant outcomes and outcome domains. Because it was difficult for patients to respond readily to the notion of outcome, analysts concentrated on going beyond a simple cataloguing of outcomes to form a deeper understanding of what participants wanted and expected from a medication review. They each worked individually, then compared their findings and reached consensus on a final report. Both analysts were experienced in qualitative research and blind to the systematic review findings. In a later stage, the main researcher (JBB), who performed the systematic review and led the development of the COS, reviewed the analysis reports and read the transcripts. The three researchers met to discuss findings, and any disagreement was resolved by discussion and consensus. The results, including consensus achieved by the two researchers and unsolved disagreements, were further discussed with healthcare professionals and other researchers to validate a final list of eligible outcomes for the Delphi survey.

Next, the results from the systematic review and the qualitative study were merged into a list of outcomes during a consultation exercise. Clinical experts and researchers identified overlap between outcomes and checked the medical and the plain language terms. People without medical knowledge further improved the plain language terms. The outcomes were classified into domains and areas according to the OMERACT classification [29]. The plain language terms and explanations were tested for understandability with two older patients (aged 86 and 92) and thereby improved. This provided a final list of outcomes and definitions.

Outcomes were selected regardless of feasibility issues during the systematic review, qualitative study, and consultation exercise. The purpose was to identify all possibly relevant outcomes, particularly for patients. If a feasibility issue was stressed by participants during the Delphi survey, this issue was discussed during consensus meetings until consensus was achieved.

### Delphi questionnaire survey

Three groups of stakeholders were drawn (164 individuals). Participants in each group were recruited in the four European countries by the medical centers, and additional external participants were recruited worldwide for Group 3. Group 1 consisted of family caregivers, patients aged 65 to 80, and patients older than 80. Group 2 consisted of healthcare professionals: general practitioners (GPs), community and hospital pharmacists, geriatricians, specialists in internal medicine, and nurses. Group 3 (the ’expert’ group) included medication review researchers (recruited based on their publication’s profile, assessed via Scopus®) and researchers in other relevant areas (e.g. sociologists of aging), representatives of scientific organizations, and policymakers. We aimed for proportions of 35%, 35%, and 30% of participants from Groups 1, 2, and 3, respectively. Participants in Group 1 were identified from researchers’ professional networks, local associations for older people, and personal networks. In order to ensure enough variability among patients, we purposively selected participants who varied in terms of age, gender, and practice setting. Participants in Group 2 were recruited among healthcare professionals from the academic hospital, other secondary non-academic hospitals, ambulatory care centers, and nursing homes connected or not to the local coordinating center.

The online questionnaire was developed by a company (WorldAPP®) specializing in online surveys (see http://app.keysurvey.fr/f/1038815/5ded/).^1^ We expected completion of the online survey to be difficult for some older patients. All centers therefore proposed to older patients or family caregivers to complete the questionnaire with the help of a local interviewer at home or during a consultation. The other stakeholders answered it online.

The Delphi process consisted of three rounds of questionnaires. Participants who did not participate in Round 1 were not invited for Round 2, and those who did not participate in Round 2 were not invited for Round 3 [33]. In Rounds 1 and 2, participants were asked to score each outcome on a scale from 1 (not important) to 9 (of critical importance). Outcomes considered as ’very important’ (rating 7 to 9) by ≥ 75% of participants in at least one stakeholder group were presented in the subsequent Delphi round. Additional outcomes suggested by Round 1 participants were presented in Round 2, if relevant. In Round 2, responses from Round 1 were summarized and were presented to the participants with their own results and the mean results of each stakeholder group. In Round 3, all participants were asked whether the outcomes should be systematically measured in all studies of medication review in the elderly (YES/NO). An outcome was eligible for inclusion in the COS if ≥ 75% of participants in at least one stakeholder group rated it YES in Round 3.

### Protocol adaptations

During the study, two deviations from the original protocol were needed. In the first, outcomes that did not meet the consensus rating for inclusion (i.e. ≥ 75% rating of 7 to 9 in at least one stakeholder group) were removed at each stage. The original protocol rule was to remove outcomes that met a consensus rating for exclusion (i.e. ≥ 75% rating of 1 to 3 in all groups). This change was motivated by high ratings by participants, comments of participants who felt the questionnaire was too long, and those of local interviewers who reported that most older participants felt tired at the end of the first questionnaire (Round 1). In the second deviation, the rating scale was replaced by a direct YES/NO question in Round 3 to further limit the number of outcomes in the final COS. Both deviations were discussed with participants during consensus meetings and with external experts and were approved by the steering committee.

### Consensus meetings

After Round 1 and Round 2, feedback was retrieved by local interviewers for older patients and by comments made in the online questionnaire by some other patients, family caregivers, healthcare professionals, and the experts. Most comments made by the experts were very clear and precise. All comments were summarized and discussed during two consensus meetings to agree on the list of outcomes for the next round: (1) with healthcare professionals from the Belgian center and (2) with the steering committee. Final decisions were taken by the steering committee.

After Round 3, three consensus meetings took place, including a face-to-face meeting with patients and caregivers in Belgium, a conference call with healthcare professionals from the Belgian center, and a conference call with medication review experts and the steering committee members. Due to language issues, patients and family caregivers from other centers were not invited. The results of each round were presented, with the consensus results from Round 3 analyses used as the starting point for discussion. The goal was to comprehensively address points for discussion and to agree on the final COS. Interaction between participants from the three groups was indirect: the feedback and opinions of each were transmitted to the others by the coordinators (JBB and AS). A final consensus meeting was held by conference call for the steering committee members and primary local investigators of the OPERAM project.

## Results

### List of eligible outcomes

The systematic review identified 57 eligible outcomes from 47 published studies and 32 RCT protocols (see Additional file 2: Additional references). Fifteen interviews were conducted with patients and family caregivers. The interpretative approach identified 19 outcomes, seven of which had not been identified in the systematic review. A final list of 64 (57 + 7) eligible outcomes was obtained by merging the two lists. These outcomes were grouped into eight health domains according to the recommendations of the OMERACT Filter 2.0 (Additional file 3: Table S2).

### Delphi survey

Of the 164 participants invited, 150 completed Round 1, including 55 patients or family caregivers, 55 healthcare professionals, and 40 experts. The participant characteristics are detailed in Additional file 4: Table S3. Nearly half the patients were older than 80, and more than one third of the healthcare professionals were GPs. The four centers recruited 126 participants, divided evenly between Belgium (*n* = 33, 26%), Ireland (*n* = 30, 24%), the Netherlands (*n* = 32, 25%), and Switzerland (*n* = 31, 25%). The 24 remaining participants were specialized medication review researchers from Australia (*n* = 3), Europe (*n* = 13), and North America (*n* = 8).

**Fig. 1:**
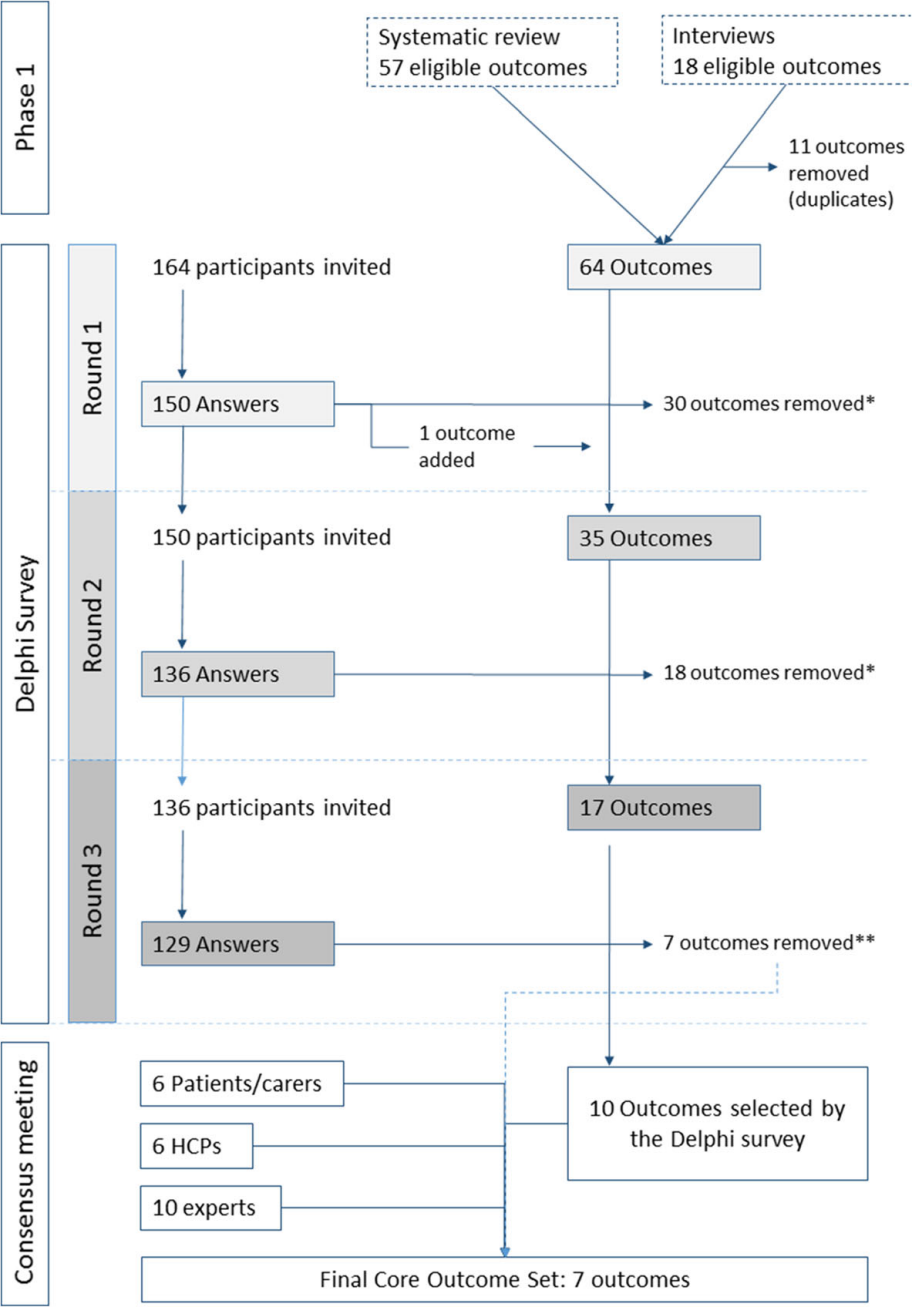
Diagram showing the flow of participants and outcomes during the three rounds of the Delphi survey and the last consensus meeting

The flow of participants and outcomes is presented in Fig. 1. Of the 150 who completed Round 1, 136 completed Round 2, and 129 completed Round 3. The overall attrition rate was 14%. After Round 1, 31 outcomes did not meet the consensus rating for inclusion in any of the three groups, including the outcome ’death’. Participants proposed 32 other outcomes. Most overlapped with outcomes proposed during Round 1. One outcome expanded the concept of treatment burden, which was split into two distinct outcomes: ’drug regimen complexity’ and ’number of drugs taken daily’. The consensus meeting decided to remove 30 outcomes; ’death’ was kept for Round 2 at the request of two stakeholder groups, and the ’drug regimen complexity’ outcome was added. Thirty-five outcomes were presented to the participants in Round 2.

After Round 2, 18 outcomes did not meet the consensus rating for inclusion in any of the three groups, including ’death’, and these outcomes were therefore removed. The consensus meeting decided to keep 17 outcomes in Round 3. The direct question in Round 3 (YES/NO) led to the selection of 10 outcomes, presented in Table 1. Two were selected by all three groups: ’drug-related hospital admissions’ and ‘serious adverse drug events’.

**Table 1:** Results of Round 3 of the Delphi survey and of the consensus meetings

Domain	Outcome^a^	Delphi survey, Round 3			Consensus meetings	
		(% answering YES)^b^				
		Patients	HCPs	Experts	Discussion required before final inclusion	Final decision
Medication use	Unnecessary drugs (overuse)	69	**76**	**97**		IN
	Underuse	70	73	**83**		IN
	Clinically significant drug-drug interaction	**100**	**92**	71		IN
	Suitability of drug dosage according to renal function	70	**88**	57	Yes	Feasibility issue
	Potentially inappropriate medications	55	68	68	Yes	IN
	Measurement of appropriateness	**80**	60	**75**	Yes	OUT
	Compliance with medication	62	**76**	67	Yes	OUT
Use of healthcare resources	Hospitalization (all causes of hospitalization)	62	55	35		OUT
Adverse events	Drug-related hospital admissions	**83**	**86**	**84**		IN
	Adverse drug reaction	70	67	57		OUT
	Serious adverse drug reaction	**98**	**94**	**77**	Yes	Feasibility issue
	Adverse drug withdrawal event	68	68	57		OUT
Patient-reported outcomes	Quality of life	**93**	71	67		IN
	Pain relief	**89**	49	23	Yes	IN
Process evaluation	Elicitation and consideration of patient preference	65	69	56		OUT
	Level of satisfaction of the patient’s GP with communication on medication change	61	65	37		OUT
	Patient’s satisfaction with the information on his/her medication treatment	69	60	62		OUT

^a^Based on the 17 outcomes selected after Round 2

^b^**Boldface** indicates ≥ 75% YES ratings

Abbreviation: *HCP* healthcare provider

### International consensus

Of the 11 patients or family caregivers who participated in Round 3 in Belgium, six agreed to participate in a face-to-face consensus meeting. All were women: three aged 65 to 80 and three older than 80. Of the 14 healthcare professionals who participated in Round 3 in Belgium, six (three GPs, one hospital physician, and two pharmacists) agreed to participate in a conference call consensus meeting. Due to limited availability, no researcher attended the consensus meeting. However, three specialized medication review researchers agreed to participate in a one-hour conference call to discuss the results.

The comments and consensus obtained during the meetings and conference calls were unambiguous for 11 of the 17 outcomes reviewed. Four were definitively included and seven removed. The six remaining outcomes were discussed before consensus and a final decision were reached (Table 1). For example, 89% of participants in the patient group had selected ’pain relief’ for inclusion in the COS, whereas 77% of participants in the expert group had rejected it. After discussion, a consensus was reached on including this outcome in the final COS. A summary of the discussions for these six outcomes is given in Additional file 5: Table S4.

### Final core outcome set

The final COS, with seven outcomes, is presented in Table 2. Three domains are covered: adverse events (one outcome), medication use (four outcomes), and patient-reported outcomes (two outcomes). Two other outcomes were considered highly relevant but were not included in the final COS because of feasibility issues: ’serious adverse drug reaction’ and ’suitability of drug dosage according to renal function’ (Additional file 5: Table S4).

**Table 2:** Final core outcome set with definitions of the outcomes

Domain	Outcome	Definition
Adverse events	Drug-related hospital admissions	Hospitalization due to an adverse drug event: harm due to an adverse drug reaction or a medical error related to overuse, underuse, or misuse of prescription and non-prescription medications and which is the main reason for or contributes to hospital admission of a patient
Medication use	Overuse	The use or prescription of more drugs than clinically needed, including (1) any drug prescribed or used without an evidence-based clinical indication; (2) therapeutic duplication; (3) medication prescribed or used beyond the recommended duration
	Underuse	A failure to prescribe drugs that are indicated, including (1) omission of an evidence-based drug; (2) too short a duration
	Potentially inappropriate medications	Drugs with risk of adverse drug reactions exceeding their expected clinical benefit to patients, particularly when safer therapeutic alternatives are available to treat the same condition [40]
	Clinically significant DDI	A clinically significant DDI is defined as having a significant severity rating according to the drug interaction compendia used in the study (e.g. *Drug Interaction Facts* or *Micromedex*) [41]
Patient-reported outcomes	Health-related quality of life	Personal health status: HRQoL usually refers to aspects of our lives that are dominated or significantly influenced by our mental or physical well-being
	Pain relief	Whether pain has improved over the course of the trial

Abbreviations: *HRQoL* Health-related quality of life, *DDI* drug-drug interaction

## Discussion

### Summary

This study aimed to identify outcomes of great relevance to clinical trials of medication review in multi-morbid older patients with polypharmacy from different stakeholders’ perspectives. For this purpose, the study used methodological guidance of initiatives like COMET and OMERACT [21, 29] and a Delphi survey of 129 participants from four European countries along with experts from three continents. The opinions of old and very old patients, healthcare professionals, and experts were acknowledged equally, a feature lacking in most COS research. Consensus was achieved on seven outcomes to include in the COS, which can be recommended for future use in clinical trials.

### Discussion of the included and excluded outcomes

#### Domain: adverse events

The two outcomes with the highest degree of agreement, ‘drug-related hospital admissions’ and ‘serious adverse drug reaction’, were related to the adverse events domain. More than 75% of participants in all three stakeholder groups selected these outcomes. The ’serious adverse drug reaction’ outcome could not be included in the final COS because its measurement outside the hospital environment has seldom been investigated [34] and would not be feasible in all RCTs on medication review. Moreover, drug-related hospital admission represents one component of serious adverse drug reactions, leading to an overlap between the two outcomes. Although reducing drug-related adverse events is a major goal of medication reviews, this outcome was poorly investigated in previous RCTs. In a systematic review, only 7 of 47 studies reported data on drug-related admissions [32]. Serious adverse drug reactions were reported in only one of the 47 studies, probably because of the feasibility issue [35].

#### Domain: medication use

Four of the seven outcomes selected in the final COS relate to medication use: ‘overuse’, ‘underuse’, ‘potentially inappropriate medications’, and ‘clinically significant drug-drug interactions’. Together these outcomes cover the concept of appropriateness of prescriptions. The effect of a medication review on the appropriate use of medication in older patients has mostly been evaluated through more global measurement tools such as drug-related problems or the Medication Appropriateness Index [7, 17, 32]. Underuse, overuse, and clinically significant drug-drug interactions were reported as separate measures in only 6%, 6%, and 9%, respectively, of prospective studies on medication review in older patients [32]. Interestingly, the number of drugs was not considered a very important outcome by any group and did not qualify for Round 3. Quality, it seems, matters more than quantity.

#### Domain: patient-reported outcomes

Two of the seven outcomes in the final COS were patient-reported outcomes. There was a clear consensus among stakeholder groups on including ‘health-related quality of life’. This outcome has been reported in numerous published studies of medication review in older patients [17, 19, 32]. However, the inclusion of ‘pain relief’ in the COS was more controversial. This outcome had never been investigated in previous studies and was identified during the qualitative study. It may be argued that it is only pertinent to a small proportion of patients. However, chronic pain symptoms affect more than half of all people aged ≥ 65 and are associated with negative clinical outcomes, and most older patients with chronic pain syndromes take analgesics [36–38]. More than 75% of older patients who participated in the Delphi survey saw this outcome as very important in all three rounds. The consensus meeting concluded that we cannot ask older patients their opinions and then reject the results when they do not correspond with experts’ views [39].

### Implications for future research

Effectiveness of medication review in older patients has most often been evaluated through outcomes related to healthcare use and mortality, such as all-cause hospitalizations, length of hospital stay, emergency department visits, and all-cause deaths [11, 15, 16, 20]. These outcomes were not considered essential by most stakeholders in our Delphi survey, including older patients. This result highlights the importance of developing a COS that avoids reductive conclusions based on outcomes selected by researchers only. Most outcomes included in our COS have been underreported in previous RCTs of medication review. We contend, therefore, that efforts should be made in the future to provide data on these outcomes, to determine more comprehensively the effectiveness of medication review in older patients. We did not ask participants to consider the feasibility of measuring the outcomes during the Delphi survey. Future research should determine which measurement tool is suitable for each outcome.

### Strengths and limitations

This is the first Delphi study to explore international, multi-stakeholder, and multidisciplinary consensus on core outcomes to be reported in clinical trials of medication review in older patients with multi-morbidity and polypharmacy. The strengths of this study include the following: (1) use of methods following the guidance of initiatives like COMET and OMERACT [21, 29]; (2) the large number of older and very old patients involved; (3) interviews with very old patients to increase participation; (4) a large expert panel of stakeholders representing various disciplines and countries; (5) giving participants the opportunity to comment on each choice; (6) low attrition rate; (7) rigorous reporting of methods and results according to COS-STAR guidelines [30].

Our study has some limitations. Departing from the protocol, we had to adapt the methods which were used to obtain consensus during the study. These changes were discussed and validated with the steering committee, the OPERAM research team, and two external experts in COS development. This was in line with the existing guidelines for COS development, which do not recommend one specific method but state that several methods can be used and mixed if necessary to achieve consensus according to the specific needs of the study [21, 29]. Moreover, Delphi surveys are flexible, with various recognized ways of measuring consensus [33]. Our choices were made in order to combine scientific rigor with pragmatism and relevance. Finally, patients and caregivers included in the qualitative study and in the consensus meeting were only from Belgium. The health-seeking culture and attitudes towards polypharmacy may vary across countries, and some comments or consensus achieved by Belgian participants may be specific to their culture or the Belgian health organization. However, the Delphi study included numerous patients and healthcare professionals from the four countries involved in the study, in addition to experts from several countries, which implies a real international perspective in the development of this COS.

## Conclusions

A consensus-based COS for medication review in older patients with multi-morbidity and polypharmacy was developed and included the following seven outcomes: (1) drug-related hospital admissions; (2) drug overuse; (3) drug underuse; (4) potentially inappropriate medications; (5) clinically significant drug-drug interactions; (6) health-related quality of life; (7) pain relief. A COS will only have an impact if it is consistently implemented in most trials of relevance in any given area of research. Trialists, regulators, funding agencies, and journals publishing clinical trials in the area of multi-morbidity and polypharmacy in late life should, we believe, aim to ensure that the appropriate COS is used. Further research should establish which instruments are the most appropriate for measuring these core outcomes.

## Additional files

Additional file 1: Table S1. COS-STAR checklist as recommended by Kirkham et al. [30]. (PDF 162 kb)

Additional file 2: Additional references: References of the 47 published studies and 32 RCT protocols identified in the systematic review. (PDF 107 kb)

Additional file 3: Table S2. Names of the health domains and subdomains used to classify the outcomes extracted from the systematic review and the qualitative study, according to the OMERAT Filter 2.0 classification. (PDF 96 kb)

Additional file 4: Table S3. Characteristics of the participants in the Delphi survey (all three rounds). (PDF 241 kb)

Additional file 5: Table S4. Specific decisions during the consensus meetings after Round 3. The table gives the elements of discussion and consensus obtained for six outcomes for which discrepancies were observed between stakeholder groups in the Delphi survey or for which a feasibility issue was raised by the participants. (PDF 77 kb)

## Abbreviations

COMET: Core Outcome Measures in Effectiveness Trials
COS: Core outcome set
COS-STAR: Core Outcome Set-STAndards for Reporting
OMERACT: Outcome Measures in Rheumatology
RCT: Randomized controlled trial

## Footnotes

1 In this online test version, the user chooses the language, participant category, and age group before accessing the questionnaire. During the Delphi survey, these questions were pre-filled and hidden. The participants had direct access to the questionnaire in the right form and the appropriate language.
