# Supplementary material for: International core outcome set for clinical trials of medication review in multi-morbid older patients with polypharmacy
Source: BMC Med. 2018 Feb 13;16:21. doi: 10.1186/s12916-018-1007-9 (PMC5809844; doi:10.1186/s12916-018-1007-9)
Supplement: Supplementary file 1 — COS-STAR checklist as recommended by Kirkham et al. [30]. (PDF 162 kb) [file 12916_2018_1007_MOESM1_ESM.pdf]

S-Table 1: COS-STAR checklist as recommended by Kirkham et al.[30]

SECTION/TOPIC	CHECKLIST ITEM	Location
**TITLE/ABSTRACT**		
Title	Identify in the title that the paper reports the development of a COS	Title page
Abstract	Provide a structured summary	Abstract
**INTRODUCTION**		
Background and objectives	Describe the background and explain the rationale for developing the COS	Introduction; §1
	Describe the specific objectives with reference to developing a COS	Introduction; §2
Scope	Describe the health condition(s) and population(s) covered by the COS	Introduction; §3
	Describe the intervention(s) covered by the COS	Introduction; §3
	Describe the setting(s) in which the COS is to be applied	Introduction; §3
**METHODS**		
Protocol/Registry Entry	Indicate where the COS development protocol can be accessed, if available and/or the study registration details	Methods; "Study design" part.
Participants	Describe the rationale for stakeholder groups involved in the COS development process, eligibility criteria for participants from each group and a description of how the individuals involved were identified	Methods; "Delphi questionnaire survey" part.
Information sources	Describe the information sources used to identify an initial list of outcomes	Methods; "List of potential outcomes for inclusion in Delphi survey" part
	Describe how outcomes were dropped/combined, with reasons (if applicable)	Methods; "List of potential outcomes for inclusion in Delphi survey" part
Consensus process	Describe how the consensus process was undertaken	Methods; "Delphi questionnaire survey" part.
Outcome scoring	Describe how outcomes were scored and scores summarised	Methods; "Delphi questionnaire survey" part.
Consensus definition	Describe the consensus definition	Methods; "Delphi questionnaire survey" part.
	Describe the procedure for determining how outcomes were included or excluded from consideration during the consensus process	Methods; "Delphi questionnaire survey" and "Consensus meetings" parts.
Ethics and consent	Provide a statement regarding the ethics and consent issues for the study	Methods; "Ethical approval" part

**RESULTS**		
Protocol deviations	Describe any changes from the protocol (if applicable), with reasons, and a describe what impact these changes have on the results	Methods; "Protocol adaptations" part.
Participants	Present data on the number and relevant characteristics of the people involved at all stages of COS development	Results; "Delphi survey" part
Outcomes	List all outcomes considered at the start of the consensus process	Supplementary data; S-Table 2
	Describe any new outcomes introduced and any outcomes dropped, with reasons, during the consensus process	Results "Delphi survey" and "international consensus" parts; Table 1; S-Table 4
Core outcome set	List the outcomes in the final core outcome set	Table 2
**DISCUSSION**		
Limitations	Discuss any limitations in the COS development process	Discussion section
Conclusions	Provide an interpretation of the final COS in the context of other evidence, and implications for future research	Discussion section
**OTHER INFORMATION**		
Funding	Describe sources of funding, role of funders	Acknowledgments
Conflicts of interest	Describe any conflicts of interest within the study team and how these were managed	Conflict of interest
