# Supplementary material for: International core outcome set for clinical trials of medication review in multi-morbid older patients with polypharmacy
Source: BMC Med. 2018 Feb 13;16:21. doi: 10.1186/s12916-018-1007-9 (PMC5809844; doi:10.1186/s12916-018-1007-9)
Supplement: Supplementary file 2 — Additional references: References of the 47 published studies and 32 RCT protocols identified in the systematic review. (PDF 107 kb) [file 12916_2018_1007_MOESM2_ESM.pdf]

**Additional references:** References of the 47 published studies [1–47] and 32 RCT protocols [48–79] identified in the systematic review.

1. Gillespie U, Alassaad A, Henrohn D, Garmo H, Hammarlund-Udenaes M, Toss H, et al. A comprehensive pharmacist intervention to reduce morbidity in patients 80 years or older: a randomized controlled trial. Arch. Intern. Med. 2009;169:894–900.

2. Mortimer C, Emmerton L, Lum E. The impact of an aged care pharmacist in a department of emergency medicine. J. Eval. Clin. Pract. 2011;17:478–85.

3. Spinewine A, Swine C, Dhillon S, Lambert P, Nachega JB, Wilmotte L, et al. Effect of a collaborative approach on the quality of prescribing for geriatric inpatients: a randomized, controlled trial. J. Am. Geriatr. Soc. 2007;55:658–65.

4. Lisby M, Thomsen A, Nielsen LP, Lyhne NM, Breum-Leer C, Fredberg U, et al. The effect of systematic medication review in elderly patients admitted to an acute ward of internal medicine. Basic Clin. Pharmacol. Toxicol. 2010;106:422–7.

5. Hellström LM, Bondesson A, Höglund P, Midlöv P, Holmdahl L, Rickhag E, et al. Impact of the Lund Integrated Medicines Management (LIMM) model on medication appropriateness and drug-related hospital revisits. Eur. J. Clin. Pharmacol. 2011;67:741–52.

6. Browne C, Kingston C, Keane C. Falls prevention focused medication review by a pharmacist in an acute hospital: implications for future practice. Int. J. Clin. Pharm. 2014;36:969–75.

7. Bergqvist M, Ulfvarson J, Karlsson EA. Nurse-led medication reviews and the quality of drug treatment of elderly hospitalized patients. Eur. J. Clin. Pharmacol. 2009;65:1089–96.

8. Schmader KE, Hanlon JT, Pieper CF, Sloane R, Ruby CM, Twersky J, et al. Effects of geriatric evaluation and management on adverse drug reactions and suboptimal prescribing in the frail elderly. Am. J. Med. 2004;116:394–401.

9. Gallagher P, O'Connor M, O'Mahony D. Prevention of Potentially Inappropriate Prescribing for Elderly Patients: A Randomized Controlled Trial Using STOPP/START Criteria. Clin. Pharmacol. Ther. 2011;89:845–54.

10. Krska J, Cromarty JA, Arris F, Jamieson D, Hansford D, Duffus PR, et al. Pharmacist-led medication review in patients over 65: a randomized, controlled trial in primary care. Age Ageing. 2001;30:205–11.

11. Nazareth I, Burton A, Shulman S, Smith P, Haines A, Timberal H. A pharmacy discharge plan for hospitalized elderly patients--a randomized controlled trial. Age Ageing. 2001;30:33–40.

12. Zermansky AG, Petty DR, Raynor DK, Freemantle N, Vail A, Lowe CJ. Randomised controlled trial of clinical medication review by a pharmacist of elderly patients receiving repeat prescriptions in general practice. BMJ. 2001;323:1340–3.

13. Sellors J, Kaczorowski J, Sellors C, Dolovich L, Woodward C, Willan A, et al. A randomized controlled trial of a pharmacist consultation program for family physicians and their elderly patients. CMAJ Can. Med. Assoc. J. J. Assoc. Medicale Can. 2003;169:17–22.

14. Naunton M, Peterson GM. Evaluation of Home-Based Follow-Up of High-Risk Elderly Patients Discharged from Hospital. J. Pharm. Pract. Res. 2003;33:176–82.

15. Sturgess IK, McElnay JC, Hughes CM, Crealey G. Community pharmacy based provision of pharmaceutical care to older patients. Pharm. World Sci. PWS. 2003;25:218–26.

16. Holland R, Lenaghan E, Harvey I, Smith R, Shepstone L, Lipp A, et al. Does home based medication review keep older people out of hospital? The HOMER randomised controlled trial. BMJ. 2005;330:293.

17. Lenaghan E, Holland R, Brooks A. Home-based medication review in a high risk elderly population in primary care--the POLYMED randomised controlled trial. Age Ageing. 2007;36:292–7.

18. Fiss T, Dreier A, Meinke C, van den Berg N, Ritter CA, Hoffmann W. Frequency of inappropriate drugs in primary care: analysis of a sample of immobile patients who received periodic home visits. Age Ageing. 2011;40:66–73.

19. Bryant LJM, Coster G, Gamble GD, McCormick RN. The General Practitioner-Pharmacist Collaboration (GPPC) study: a randomised controlled trial of clinical medication reviews in community pharmacy. Int. J. Pharm. Pract. 2011;19:94–105.

20. Elliott RA, Martinac G, Campbell S, Thorn J, Woodward MC. Pharmacist-led medication review to identify medication-related problems in older people referred to an Aged Care Assessment Team: a randomized comparative study. Drugs Aging. 2012;29:593–605.

21. Furniss L, Burns A, Craig SK, Scobie S, Cooke J, Faragher B. Effects of a pharmacist's medication review in nursing homes. Randomised controlled trial. Br. J. Psychiatry J. Ment. Sci. 2000;176:563–7.

22. Roberts MS, Stokes JA, King MA, Lynne TA, Purdie DM, Glasziou PP, et al. Outcomes of a randomized controlled trial of a clinical pharmacy intervention in 52 nursing homes. Br. J. Clin. Pharmacol. 2001;51:257–65.

23. King MA, Roberts MS. Multidisciplinary case conference reviews: improving outcomes for nursing home residents, carers and health professionals. Pharm. World Sci. PWS. 2001;23:41–5.

24. Crotty M, Halbert J, Rowett D, Giles L, Birks R, Williams H, et al. An outreach geriatric medication advisory service in residential aged care: a randomised controlled trial of case conferencing. Age Ageing. 2004;33:612–7.

25. Zermansky AG, Alldred DP, Petty DR, Raynor DK, Freemantle N, Eastaugh J, et al. Clinical medication review by a pharmacist of elderly people living in care homes--randomised controlled trial. Age Ageing. 2006;35:586–91.

26. Patterson SM, Hughes CM, Crealey G, Cardwell C, Lapane KL. An evaluation of an adapted U.S. model of pharmaceutical care to improve psychoactive prescribing for nursing

home residents in northern ireland (fleetwood northern ireland study). J. Am. Geriatr. Soc. 2010;58:44–53.

27. Pope G, Wall N, Peters CM, O'Connor M, Saunders J, O'Sullivan C, et al. Specialist medication review does not benefit short-term outcomes and net costs in continuing-care patients. Age Ageing. 2011;40:307–12.

28. Lapane KL, Hughes CM, Daiello LA, Cameron KA, Feinberg J. Effect of a pharmacist-led multicomponent intervention focusing on the medication monitoring phase to prevent potential adverse drug events in nursing homes. J. Am. Geriatr. Soc. 2011;59:1238–45.

29. Milos V, Rekman E, Bondesson Å, Eriksson T, Jakobsson U, Westerlund T, et al. Improving the quality of pharmacotherapy in elderly primary care patients through medication reviews: a randomised controlled study. Drugs Aging. 2013;30:235–46.

30. Blalock SJ, Casteel C, Roth MT, Ferreri S, Demby KB, Shankar V. Impact of enhanced pharmacologic care on the prevention of falls: a randomized controlled trial. Am. J. Geriatr. Pharmacother. 2010;8:428–40.

31. Meredith S, Feldman P, Frey D, Giammarco L, Hall K, Arnold K, et al. Improving medication use in newly admitted home healthcare patients: a randomized controlled trial. J. Am. Geriatr. Soc. 2002;50:1484–91.

32. Dalleur O, Boland B, Losseau C, Henrard S, Wouters D, Speybroeck N, et al. Reduction of potentially inappropriate medications using the STOPP criteria in frail older inpatients: a randomised controlled study. Drugs Aging. 2014;31:291–8.

33. Trygstad TK, Christensen D, Garmise J, Sullivan R, Wegner S. Pharmacist response to alerts generated from Medicaid pharmacy claims in a long-term care setting: results from the North Carolina polypharmacy initiative. J. Manag. Care Pharm. JMCP. 2005;11:575–83.

34. Pit SW, Byles JE, Henry DA, Holt L, Hansen V, Bowman DA. A Quality Use of Medicines program for general practitioners and older people: a cluster randomised controlled trial. Med. J. Aust. 2007;187:23–30.

35. Kwint HF, Faber A, Gussekloo J, Bouvy ML. Effects of medication review on drug-related problems in patients using automated drug-dispensing systems: a pragmatic randomized controlled study. Drugs Aging. 2011;28:305–14.

36. Gnjidic D, Le Couteur DG, Abernethy DR, Hilmer SN. A Pilot Randomized Clinical Trial Utilizing the Drug Burden Index to Reduce Exposure to Anticholinergic and Sedative Medications in Older People. Ann. Pharmacother. 2010;44:1725–32.

37. Touchette DR, Masica AL, Dolor RJ, Schumock GT, Choi YK, Kim Y, et al. Safety-focused medication therapy management: a randomized controlled trial. J. Am. Pharm. Assoc. JAPhA. 2012;52:603–12.

38. Frankenthal D, Lerman Y, Kalendaryev E, Lerman Y. Intervention with the Screening Tool of Older Persons Potentially Inappropriate Prescriptions/Screening Tool to Alert Doctors to Right Treatment Criteria in Elderly Residents of a Chronic Geriatric Facility: A Randomized Clinical Trial. J. Am. Geriatr. Soc. 2014;62:1658–65.

39. Ahmad A, Nijpels G, Dekker JM, Kostense PJ, Hugtenburg JG. EFfect of a pharmacist medication review in elderly patients discharged from the hospital. Arch. Intern. Med. 2012;172:1346–7.

40. Lisby M, Bonnerup DK, Brock B, Gregersen PA, Jensen J, Larsen M-L, et al. Medication Review and Patient Outcomes in an Orthopedic Department: A Randomized Controlled Study. J. Patient Saf. 2015;

41. Chan D-C, Chen J-H, Wen C-J, Chiu L-S, Wu S-C. Effectiveness of the medication safety review clinics for older adults prescribed multiple medications. J. Formos. Med. Assoc. Taiwan Yi Zhi. 2014;113:106–13.

42. Jódar-Sánchez F, Malet-Larrea A, Martín JJ, García-Mochón L, López Del Amo MP, Martínez-Martínez F, et al. Cost-utility analysis of a medication review with follow-up service for older adults with polypharmacy in community pharmacies in Spain: the conSIGUE program. PharmacoEconomics. 2015;33:599–610.

43. Lenander C, Elfsson B, Danielsson B, Midlöv P, Hasselström J. Effects of a pharmacist-led structured medication review in primary care on drug-related problems and hospital admission rates: a randomized controlled trial. Scand. J. Prim. Health Care. 2014;32:180–6.

44. Mestres C, Hernandez M, Llagostera B, Espier M, Chandre M. Improvement of pharmacological treatments in nursing homes: medication review by consultant pharmacists. Eur. J. Hosp. Pharm. 2015;ejhpharm-2014-000508.

45. O'Sullivan D, O'Mahony D, O'Connor MN, Gallagher P, Cullinan S, O'Sullivan R, et al. The impact of a structured pharmacist intervention on the appropriateness of prescribing in older hospitalized patients. Drugs Aging. 2014;31:471–81.

46. Paquin AM, Salow M, Rudolph JL. Pharmacist calls to older adults with cognitive difficulties after discharge in a Tertiary Veterans Administration Medical Center: a quality improvement program. J. Am. Geriatr. Soc. 2015;63:571–7.

47. Westberg SM, Swanoski MT, Renier CM, Gessert CE. Evaluation of the impact of comprehensive medication management services delivered posthospitalization on readmissions and emergency department visits. J. Manag. Care Spec. Pharm. 2014;20:886–93.

48. A Pilot Study to Reduce Inappropriate Anticholinergic Prescribing in the Elderly - Full Text View - ClinicalTrials.gov [Internet]. [cited 2017 Dec 5]. Available from: https://clinicaltrials.gov/ct2/show/NCT02172612

49. A Randomized Controlled Pharmacist Intervention Study to Reduce Drug-related Problems and Readmissions Among Old People With Dementia - Full Text View - ClinicalTrials.gov [Internet]. [cited 2017 Dec 5]. Available from: https://clinicaltrials.gov/ct2/show/NCT01504672

50. ACTRN12611000370909 - Deprescribing in frail older people: a randomised controlled trial [Internet]. [cited 2017 Dec 5]. Available from: https://www.anzctr.org.au/Trial/Registration/TrialReview.aspx?ACTRN=12611000370909

51. ACTRN12611000995976 - Application of a prescribing indicators tool to assist in identifying and resolving drug-related problems in older Australians - a randomized controlled trial. [Internet]. [cited 2017 Dec 5]. Available from: http://www.anzctr.org.au/TrialSearch.aspx?searchTxt=ACTRN12611000995976&isBasic=True

52. ACTRN12615000539538 - Feasibility study of the Drug Burden Index with Home Medicine Review [Internet]. [cited 2017 Dec 5]. Available from: https://www.anzctr.org.au/Trial/Registration/TrialReview.aspx?id=368523&isReview=true

53. Clinical and Economical Assessment of an Intervention to Reduce Potentially Inappropriate Medication in Polymedicated Elderly Patients - Full Text View - ClinicalTrials.gov [Internet]. [cited 2017 Dec 5]. Available from: https://clinicaltrials.gov/ct2/show/NCT02275572

54. Cooperation for Improved Pharmacotherapy in Home-dwelling Elderly People Receiving Polypharmacy - The COOP Study - Full Text View - ClinicalTrials.gov [Internet]. [cited 2017 Dec 5]. Available from: https://clinicaltrials.gov/ct2/show/NCT02379455

55. DBI - Tool for Medication Reviews in Older People - Full Text View - ClinicalTrials.gov [Internet]. [cited 2017 Dec 5]. Available from: https://clinicaltrials.gov/ct2/show/NCT02317666

56. Discontinuing Inappropriate Medication in Nursing Home Residents - Full Text View - ClinicalTrials.gov [Internet]. [cited 2017 Dec 5]. Available from: https://clinicaltrials.gov/ct2/show/NCT01876095

57. DRKS00005734 - Use of START/STOP criteria in a web-based medication review application in community-dwelling elderl [Internet]. [cited 2017 Dec 5]. Available from: http://apps.who.int/trialsearch/Trial2.aspx?TrialID=DRKS00005734

58. Effect of Person-Centred-Care on Antipsychotic Drug Use in Nursing Homes: a Cluster-randomised Trial - Full Text View - ClinicalTrials.gov [Internet]. [cited 2017 Dec 5]. Available from: https://clinicaltrials.gov/ct2/show/NCT02295462

59. Inappropriate Prescription in Elderly and Polypharmacy Patients in Primary Care (PHARM-PC) Trial - Full Text View - ClinicalTrials.gov [Internet]. [cited 2017 Dec 5]. Available from: https://clinicaltrials.gov/ct2/show/NCT02224833

60. ISRCTN - ISRCTN10137559: PRIMA-eDS: Polypharmacy in chronic diseases: Reduction of Inappropriate Medication and Adverse drug events in elderly populations by electronic Decision Support [Internet]. [cited 2017 Dec 5]. Available from: http://www.isrctn.com/ISRCTN10137559

61. ISRCTN - ISRCTN38449870: Reduction of inappropriate medication and adverse drug events in older patients [Internet]. [cited 2017 Dec 5]. Available from: http://www.isrctn.com/ISRCTN38449870?q=ISRCTN38449870&filters=&sort=&offset=1&totalResults=1&page=1&pageSize=10&searchType=basic-search

62. ISRCTN - ISRCTN41595373: WestGem study: WESTphalian study on a medication therapy management and home care based intervention under Gender specific aspects in Elderly Multimorbid patients [Internet]. [cited 2017 Dec 5]. Available from:

http://www.isrctn.com/ISRCTN41595373?q=ISRCTN41595373&filters=&sort=&offset=1&totalResults=1&page=1&pageSize=10&searchType=basic-search

63. ISRCTN - ISRCTN41694007: OptImizing prescribing for older people in primary care [Internet]. [cited 2017 Dec 5]. Available from: http://www.isrctn.com/ISRCTN41694007?q=ISRCTN41694007&filters=&sort=&offset=1&totalResults=1&page=1&pageSize=10&searchType=basic-search

64. ISRCTN - ISRCTN42003273: Polypharmacy reduction in patients treated for chronic diseases [Internet]. [cited 2017 Dec 5]. Available from: http://www.isrctn.com/ISRCTN42003273?q=ISRCTN42003273&filters=&sort=&offset=1&totalResults=1&page=1&pageSize=10&searchType=basic-search

65. ISRCTN - ISRCTN46272088: Effects of structuring doctor-patient-communication in primary care of patients with multimorbidity (MultiCare AGENDA) [Internet]. [cited 2017 Dec 5]. Available from: http://www.isrctn.com/ISRCTN46272088?q=ISRCTN46272088&filters=&sort=&offset=1&totalResults=1&page=1&pageSize=10&searchType=basic-search

66. Medication Minimization for Long-term Care Residents - Full Text View - ClinicalTrials.gov [Internet]. [cited 2017 Dec 5]. Available from: https://clinicaltrials.gov/ct2/show/NCT01932632

67. Medication Reconciliation in Comparison to an Extensive Medication Safety Check - Full Text View - ClinicalTrials.gov [Internet]. [cited 2017 Dec 5]. Available from: https://clinicaltrials.gov/ct2/show/NCT02413957

68. Medication Safety of Elderly Patients in Hospital and Ambulatory Setting - Full Text View - ClinicalTrials.gov [Internet]. [cited 2017 Dec 5]. Available from: https://clinicaltrials.gov/ct2/show/NCT01578525

69. Multidisciplinary Program "Optimization of Drug Prescription" : Impact on the Quality of Drug Prescription in Hospitalized Elderly Patients - Full Text View - ClinicalTrials.gov [Internet]. [cited 2017 Dec 5]. Available from: https://clinicaltrials.gov/ct2/show/NCT01947270

70. NTR3569 - PRescription Optimization of Psychotropic drugs in Elderly nuRsing home patients with dementia (PROPER study). [Internet]. [cited 2017 Dec 5]. Available from: http://www.trialregister.nl/trialreg/admin/rctview.asp?TC=3569

71. NTR4264 - Opti-Med: Optimisation of a medication review programme for elderly in general practice. [Internet]. [cited 2017 Dec 5]. Available from: http://www.trialregister.nl/trialreg/admin/rctview.asp?TC=4264

72. NTR4389 - The effectivity and feasibility of integrating nondispensing pharmacists into primary healthcare centres [Internet]. [cited 2017 Dec 5]. Available from: http://www.trialregister.nl/trialreg/admin/rctview.asp?TC=4389

73. NTR4895 - Evaluation of the use of a patient questionnaire in measuring patient reported outcomes of medication reviews [Internet]. [cited 2017 Dec 5]. Available from: http://www.trialregister.nl/trialreg/admin/rctview.asp?TC=4895

74. Pharmaceutical Care and Clinical Outcomes for the Elderly Taking Potentially Inappropriate Medication - Full Text View - ClinicalTrials.gov [Internet]. [cited 2017 Dec 5]. Available from: https://clinicaltrials.gov/ct2/show/NCT00844025

75. Pharmaceutical Care in VA Nursing Home - Full Text View - ClinicalTrials.gov [Internet]. [cited 2017 Dec 5]. Available from: https://clinicaltrials.gov/ct2/show/NCT01823757

76. Pharmaceutical Care of Patients With Impaired Mobility in a Model-region of Decreasing Supply of Medical Services - Full Text View - ClinicalTrials.gov [Internet]. [cited 2017 Dec 5]. Available from: https://clinicaltrials.gov/ct2/show/NCT01587599

77. Prevention of Adverse Drug Events (ADEs) in Hospitalised Older Patients - Full Text View - ClinicalTrials.gov [Internet]. [cited 2017 Dec 5]. Available from: https://clinicaltrials.gov/ct2/show/NCT01467050

78. Rationalisation of Polypharmacy by the Geriatric Consultation Team - Full Text View - ClinicalTrials.gov [Internet]. [cited 2017 Dec 5]. Available from: https://clinicaltrials.gov/ct2/show/NCT02165618

79. Rationalisation of Polypharmacy in the Elderly by the RASP Instrument - Full Text View - ClinicalTrials.gov [Internet]. [cited 2017 Dec 5]. Available from: https://clinicaltrials.gov/ct2/show/NCT01513265
