# Supplementary material for: International core outcome set for clinical trials of medication review in multi-morbid older patients with polypharmacy
Source: BMC Med. 2018 Feb 13;16:21. doi: 10.1186/s12916-018-1007-9 (PMC5809844; doi:10.1186/s12916-018-1007-9)
Supplement: Supplementary file 3 — Names of the health domains and subdomains used to classify the outcomes extracted from the systematic review and the qualitative study, according to the OMERAT Filter 2.0 classification. (PDF 96 kb) [file 12916_2018_1007_MOESM3_ESM.pdf]

S-Table 2: Names of the health domains and subdomains used to classify the outcomes extracted from the systematic review and the qualitative study, according to the OMERAT filter 2.0 classification

Core Areas	Domains	Subdomains	Source
Patho-physiological manifestations	Medication use	Number of drugs	SR + QS
		Unnecessary drugs (Overuse)	SR + QS
		Underuse	SR
		Renal impairment dose adjustment	SR
		Drug-drug interaction	SR
		Potentially inappropriate medications	SR + QS
		Anticholinergic drug use	SR
		Psychotropic drug use	SR
		Measurement of the appropriateness	SR
		Compliance with medication	SR
		Switch to generic drugs	QS
		Resumption of discontinued drugs during follow-up	SR
Death	Death	All-cause death	SR
Life impact	Patient reported outcomes	Ease of taking treatment for an older person (form, preparation, management plan, etc.)	SR + QS
		Patients' knowledge of their medications	SR
		Patients' beliefs about medications concerns	SR
		Patients' beliefs about medications necessity	SR
		Health-related quality of life	SR
		Physical symptoms and complaints	SR + QS
		Pain relief	QS
		Psychological symptoms	SR + QS
		Social life activities	QS
		Sleep quality	SR + QS
		Elicitation and consideration of patient preference	SR
		Patient's information about the changes in his/her treatment made during the medication review	SR + QS
		Patient satisfaction with the medication review	SR
	Geriatric syndromes	Urinary incontinence	SR
		Depression	SR
		Behavioural disorders	SR
		Dementia, cognitive disorders	SR
		Delirium	SR
		Use of physical restraint in patient with behaviour problems	SR

		Activities of Daily Living	SR
		Dizziness	SR
		Gait speed	SR
		Falls occurrence	SR
		Risk of falling	SR
		Fractures	SR
Resource use / economical impact	Use of healthcare resources	Hospitalisation (all causes hospitalisation)	SR
		Length of hospital stay	SR
		Emergency Room visits	SR
		Number of GP visits	SR
		cardiovascular morbidity	SR + QS
		Risk of going to a nursing home	SR
	Adverse events	Drug-related hospital admissions	SR
		Adverse drug reaction	SR + QS
		Serious adverse drug reaction	SR + QS
		Prescribing cascade	QS
		Adverse drug withdrawal event	SR
	Costs	Direct costs = costs of medications	SR + QS
		Waste of drugs	QS
		Indirect costs	SR
		Cost-effectiveness	SR
Others	Process evaluation	Number of drugs discontinued or with dosage changed	SR
		Number of drugs initiated	SR
		Clinical relevance of modifications proposed during the medication review	SR
		Confirmation that medication reviews have taken place	SR
		Check that the medical condition of the patient was assessed before performing the medication review	QS
		Involvement of the patient's GP in the medication review	QS
		Shared decision making process with the patient	SR
		Number of recommendations that have been accepted by the physician in charge of the patient	SR
		Barriers and facilitators for medication review in the elderly	SR
		Time spent by all healthcare professionals and healthcare teams to perform the medication review	SR
		Satisfaction of healthcare professionals with the medication review	SR

Abbreviations: SR: systematic review; QS: qualitative study
