# Supplementary material for: International core outcome set for clinical trials of medication review in multi-morbid older patients with polypharmacy
Source: BMC Med. 2018 Feb 13;16:21. doi: 10.1186/s12916-018-1007-9 (PMC5809844; doi:10.1186/s12916-018-1007-9)
Supplement: Supplementary file 4 — Characteristics of the participants in the Delphi survey (all three rounds). (PDF 241 kb) [file 12916_2018_1007_MOESM4_ESM.pdf]

S-Table 3: Characteristics of the participants in the Delphi survey (all three rounds).

	Round 1<br>No. (%)	Round 2<br>No. (%)	Round 3<br>No. (%)
**TOTAL participants**	***n*** **= 150**	***n*** **= 136**	***n*** **= 129**
**Patient participants***	***n*** **= 55**	***n*** **= 49**	***n*** **= 46**
Age (years)			
65-80	28 (54.9)	26 (56.5)	23 (53.5)
> 80	23 (45.1)	20 (43.5)	20 (46.5)
Sex			
Female	25 (56.8)	23 (59)	21 (58.3)
Male	19 (43.2)	16 (41)	15 (41.7)
Place of living			
Own home	41 (85.4)	36 (85.7)	33 (84.6)
Nursing Home	7 (14.6)	6 (14.3)	6 (15.4)
Centre			
Belgium (Brussels)	14 (25.5)	13 (26.5)	11 (23.9)
Ireland (Cork)	11 (20.0)	10 (20.5)	10 (21.7)
Netherlands (Utrecht)	14 (25.5)	13 (26.5)	12 (26.1)
Switzerland (Bern)	16 (29.1)	13 (26.5)	13 (28.3)
Relationship for carers	(n=13)	(n=12)	(n=11)
Partner	5 (38.5)	5 (41.7)	5 (45.4)
Child	3 (23.1)	3 (25.0)	2 (18.2)
Other	1 (7.7)	1 (8.3)	1 (9.1)
Missing data	4 (30.8)	3 (25.0)	3 (27.3)
**Health care professionals**	***n*** **= 55**	***n*** **= 52**	***n*** **= 50**
Age (years)			
≤ 30	9 (16.4)	9 (17.3)	9 (18)
30-40	22 (40.0)	22 (42.3)	20 (40)
41-50	16 (29.1)	13 (25.0)	13 (26)
51-64	8 (14.5)	8 (15.4)	8 (16)
Sex			
F	33 (60)	31 (59.6)	30 (60)
M	22 (40)	21 (39.4)	20 (40)
Profession			
GP	20 (36.4)	19 (36.5)	19 (38)
Hospital physician	10 (18.2)	9 (17.3)	9 (18)
Pharmacist	13 (23.6)	12 (23.1)	10 (20)
Nurse	12 (21.8)	12 (23.1)	12 (24)
Place of practice			
Community setting	30 (54.5)	28 (53.8)	27 (54)
Hospital	20 (36.4)	19 (36.5)	18 (36)
Nursing Homes	5 (9.1)	5 (9.6)	5 (10)
Centre			
Belgium (Brussels)	14 (25.5)	14 (26.9)	14 (28)
Ireland (Cork)	13 (23.6)	13 (25.0)	13 (26)
Netherlands (Utrecht)	18 (32.7)	15 (28.8)	13 (26)
Switzerland (Bern)	10 (18.2)	10 (19.2)	10 (20)

**Experts**	***n* = 40**	***n* = 36**	***n* = 33**
Age (years)			
≤ 30	2 (5.1)	2 (6)	2 (6.2)
30-40	6 (15.4)	5 (14)	4 (12.5)
41-50	8 (20.5)	7 (20)	6 (18.8)
51-64	23 (59.0)	21 (60)	20 (62.5)
Sex			
F	16 (40)	14 (38.9)	12 (36.4)
M	24 (60)	22 (60.1)	21 (63.6)
Profession			
Researchers med. Field	24 (60.0)	23 (63.9)	22 (66.7)
Researchers other field	9 (22.5)	9 (25.0)	4 (12.1)
Other	7 (17.5)	4 (11.1)	7 (21.2)
Country			
Australia	3	3	3
Belgium	6	5	5
Canada	2	2	2
France	1	1	1
Germany	1	1	1
Ireland (Cork)	8	8	7
Italy	1	1	1
Netherlands	2	2	2
Norway	1	1	1
Switzerland	5	3	2
UK	4	3	2
USA	6	6	6

* Age, sex and place of living are those of the patient, whoever (patient or caregiver) participated
