# Supplementary material for: International core outcome set for clinical trials of medication review in multi-morbid older patients with polypharmacy
Source: BMC Med. 2018 Feb 13;16:21. doi: 10.1186/s12916-018-1007-9 (PMC5809844; doi:10.1186/s12916-018-1007-9)
Supplement: Supplementary file 5 — Specific decisions during the consensus meetings after Round 3. The table gives the elements of discussion and consensus obtained for six outcomes for which discrepancies were observed between stakeholder groups in the Delphi survey or for which a feasibility issue was raised by the participants. (PDF 77 kb) [file 12916_2018_1007_MOESM5_ESM.pdf]

S-Table 4: Specific decisions during the consensus meetings after Round 3. The table gives the elements of discussion and consensus obtained for six outcomes for which discrepancies were observed between stakeholder groups in the Delphi survey, or for which a feasibility issue was raised by the participants.

**Outcome**	**Consensus meeting**	**Decision**
Serious adverse drug reaction	Definition of serious ADR contains drug-related admission. Consensus achieved: keep the two distinct outcomes in the COS.<br>Feasibility issue: Definition of serious ADR includes fatal ADR and ADR causing disability. The feasibility of a prospective detection and adjudication of such ADRs occurring outside a hospital have not been investigated. Those occurring in a hospital setting will be covered by the outcome “Drug-related hospital admission”	Highly recommended but major feasibility issue
Suitability of drug dosage according to renal function	Outcome more controversial with discrepancies between groups.<br>Issue of feasibility raised by most participants: (a) difficult to have this measurement done systematically in RCTs on medication review because follow-up is most often done by phone; (b) RF is dynamic and can change; (c) problem of its measurement during a hospitalisation	Highly recommended but major feasibility issue
Pain relief	Major disagreement between patients and experts. Long discussion in each consensus meeting.<br>Consensus achieved: outcome included into the COS with the precision that it is up to the researcher to choose to use a specific tool or to include pain evaluation into HRQoL	Inclusion
potentially inappropriate medications	Long discussions with agreement that both appropriateness and PIMs cannot be removed. MAI is time consuming and cannot be applied in all RCTs in medication review. Moreover, appropriateness is globally covered if underuse + overuse + drug-drug interaction + PIMs are evaluated.<br>Consensus achieved: inclusion of PIMs and exclusion of appropriateness	Inclusion
Measurement of the appropriateness		Exclusion
Compliance with medication	Very important but lack of standard way to measure it, difficult to measure, problem with hospitalized patients. May alter the general application of the COS.<br>Consensus achieved: very important but not mandatory.	Exclusion

Abbreviations: ADR: Adverse drug reaction; COS: Core outcome set; RCT: Randomized controlled trials; RF: Renal function; HRQoL: Health-related quality of life; MAI: Medication appropriateness index; PIM: Potentially inappropriate medication
